# Integrated Network Pharmacology and Machine Learning to Reveal the Mechanisms of Schisandrin A Against Triple‐Negative Breast Cancer

**DOI:** 10.1111/jcmm.71219

**Published:** 2026-06-01

**Authors:** Meng Xu, Yingchun Zhang, Rubing Mei, Tingting Xue, Hongning Cai, Yu Zhou, Jianfang Guo

**Affiliations:** ^1^ Department of Traditional Chinese Maternal and Child Health Hospital of Hubei Province Wuhan China; ^2^ Health Management Center Maternal and Child Health Hospital of Hubei Province Wuhan China; ^3^ Research and Development Center of Science and Technology Wuhan University Wuhan China

**Keywords:** GSK3B/IDO1/KDR/PKM, network pharmacology, prognostic model, Schisandrin a, triple‐negative breast cancer

## Abstract

This study aimed to elucidate the mechanism of action of Schisandrin A in the intervention of triple‐negative breast cancer (TNBC). Through the application of network pharmacology and the integration of targets from multiple databases, the research identified 128 overlapping targets between Schisandrin A and TNBC. From these, 36 core targets were determined via topological analysis. Utilizing the random forest survival algorithm, four prognosis‐related core targets (GSK3B, IDO1, KDR, PKM) were identified from the METABRIC database, and the model's predictive performance was validated using the GSE58812 dataset. Pan‐cancer expression analysis confirmed the aberrant expression of these four targets across various tumours. Immune infiltration analysis suggested that GSK3B and IDO1 may influence the tumour immune microenvironment. Molecular docking studies demonstrated a high binding affinity of Schisandrin A with IDO1 and PKM. In vitro cell experiments indicated that Schisandrin A inhibited the proliferation of MDA‐MB‐231 cells in a concentration‐dependent manner, induced apoptosis and caused cell cycle arrest at the S phase. Transcriptome sequencing further revealed the transcriptional changes induced by Schisandrin A, elucidating that the cell cycle and DNA replication are the primary regulatory pathways affected. In conclusion, Schisandrin A exhibits potential anti‐triple‐negative breast cancer (TNBC) effects by modulating and regulating diverse pathways, including GSK3B and IDO1, alongside its impact on the cell cycle and immune microenvironment. This study presents novel candidate drugs and therapeutic targets for the precise treatment of TNBC.

## 1 Introduction

Malignant tumours represent a significant public health challenge, posing a substantial threat to global health. In 2022, breast cancer emerged as the most prevalent malignancy in terms of both incidence and mortality among females worldwide, with an age‐standardized incidence rate of 46.8 per 100,000 individuals and an age‐standardized mortality rate of 12.7 per 100,000 individuals [1]. Within the spectrum of breast cancer subtypes, triple‐negative breast cancer (TNBC) is characterized by its aggressive nature and the lack of expression of the oestrogen receptor (ER), progesterone receptor (PR) and human epidermal growth factor receptor 2 (HER2) [2]. Despite advancements in systemic therapies, including chemotherapy and immunotherapy, patients with TNBC continue to experience poor prognoses, largely due to elevated rates of recurrence, metastasis and inherent drug resistance [3]. The considerable heterogeneity of TNBC further complicates therapeutic management, highlighting the urgent necessity for the development of novel, effective treatment strategies and a more comprehensive understanding of its molecular underpinnings.

Natural products have long been acknowledged as a crucial source of lead compounds in the development of anti‐tumour drugs, primarily due to their structural diversity and inherent multi‐target regulatory properties [4, 5, 6, 7, 8, 9]. Recently, research on the modulation of the tumour immune microenvironment (TIME) by natural products has emerged as a significant area of interest within the field of tumour immunotherapy [10, 11, 12]. As a critical microenvironmental niche influencing tumour initiation and progression, TIME comprises various components, including immune cells, stromal cells and cytokines. Importantly, the maturation and infiltration of antigen‐presenting cells (APCs) and the activation status of T cell subsets are pivotal in orchestrating anti‐tumour immune responses [13, 14, 15]. An increasing body of evidence has demonstrated that natural products can remodel the tumour immune microenvironment (TIME)—by promoting APC maturation, enhancing effector T cell infiltration and inhibiting the function of immunosuppressive cells to counteract tumour immune evasion and exert synergistic anti‐tumour effects [10, 11, 12]. This offers novel insights for the immunotherapy of triple‐negative breast cancer.

Schisandrin A (Sch A), a primary bioactive lignan derived from the traditional Chinese medicinal herb Schisandra chinensis, has been documented to possess a range of pharmacological activities, including anti‐inflammatory, antioxidant, hepatoprotective and anti‐tumour effects [16, 17]. In oncology, a growing body of evidence indicates that Sch A can inhibit cell proliferation and induce apoptosis in various malignant cell lines, such as those of lung cancer, hepatocellular carcinoma and oesophageal cancer [18, 19, 20]. Nevertheless, the therapeutic potential of Sch A against triple‐negative breast cancer and its underlying molecular mechanisms—particularly its regulatory influence on the tumour immune microenvironment—remains insufficiently investigated.

Network pharmacology, a systematic methodology that integrates bioinformatics and systems biology, facilitates a comprehensive examination of multicomponent and multi‐target interactions between natural products and diseases, thereby addressing the limitations inherent in traditional single‐target research [21]. Machine learning techniques can effectively identify core molecules associated with disease prognosis from a vast array of targets, thus enhancing the clinical relevance of research findings [22, 23]. The integration of network pharmacology with machine learning methodologies can further refine the accuracy of prognostic marker identification and augment the clinical translational potential of research outcomes. In this study, we employed a combination of network pharmacology, machine learning and in vitro experimental validation to systematically investigate the core targets and molecular pathways involved in Sch A‐mediated inhibition of triple‐negative breast cancer. This research aims to elucidate the core targets and key signalling pathways that mediate the anti‐TNBC efficacy of Sch A, thereby providing a novel theoretical foundation and strategic approaches for the clinical translation of Sch A and the targeted immunotherapy of TNBC.

## 2 Materials and Methods

### 2.1 Network Pharmacology Analysis

**FIGURE 1:**
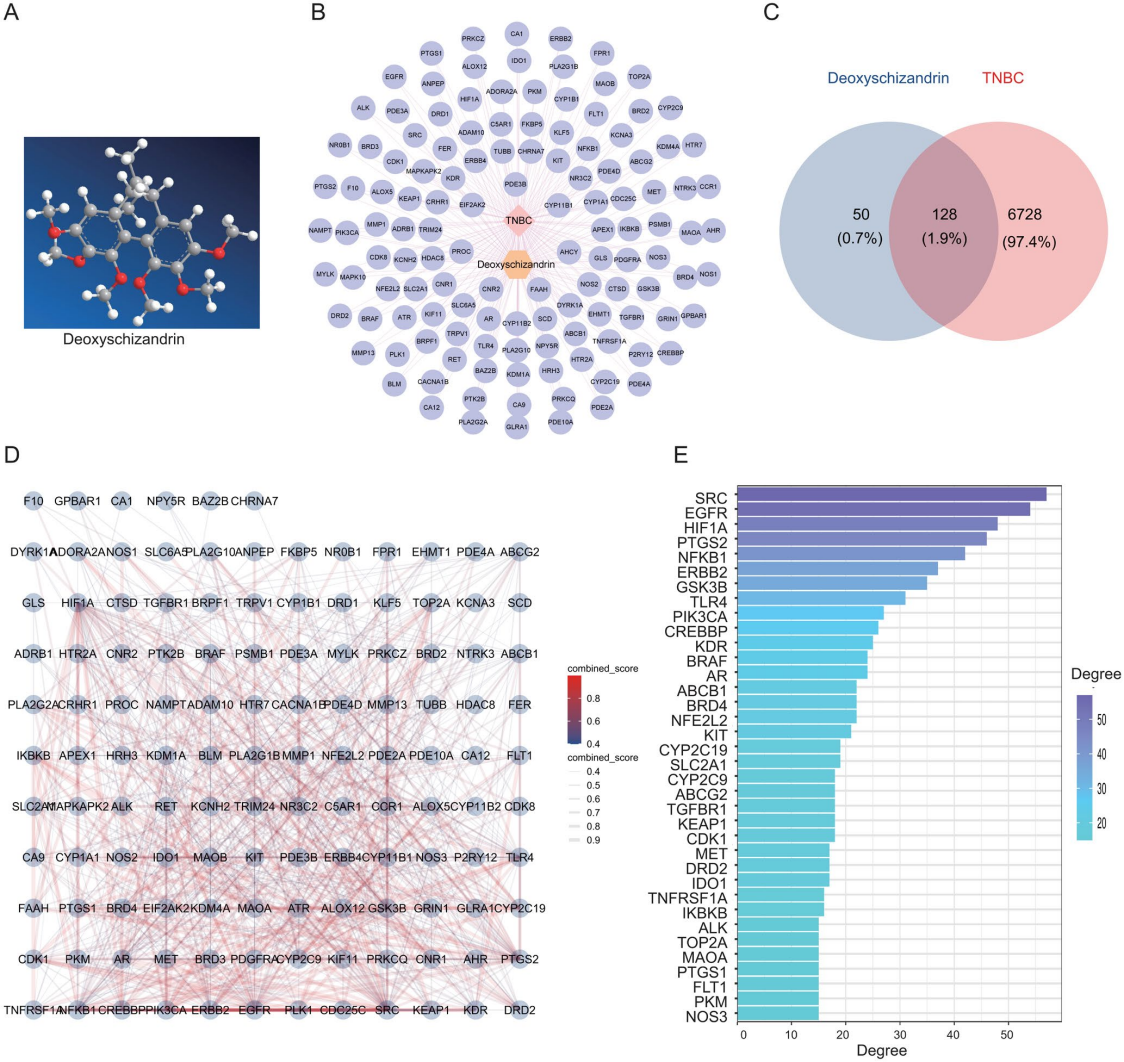
Identification of Potential Anti‐TNBC Targets of Sch A: (A) The chemical structure of Sch A is depicted. (B) A Venn diagram illustrates the intersection between potential targets of Sch A and targets associated with triple‐negative breast cancer (TNBC). (C) The overlapping region indicates 128 common targets, which are designated as potential anti‐TNBC targets of Sch A. (D) An interaction relationship diagram illustrates the intersection targets between Sch A and TNBC, with colour gradients transitioning from blue to red and line thickness indicating the magnitude of the combined score. (E) Thirty‐six genes exhibit a degree greater than 15.

Potential targets of Sch A were identified by inputting its SMILES structure (as depicted in Figure 1A) into the SEA, SWISS and Super‐PRED databases, utilizing a prediction probability threshold of ≥ 0.1. To isolate targets associated with triple‐negative breast cancer, the keyword ‘Triple‐negative breast cancer’ was employed. In the OMIM database, only targets related to human diseases were considered, while in the GeneCards database, a relevance score threshold of ≥ 20 was applied. The search, conducted in March 2025, yielded 237 targets from the OMIM database and 6762 targets from the GeneCards database. Intersection analysis between the potential drug targets of Sch A and TNBC‐related targets was performed using the Venny 2.1 tool, and a Venn diagram was generated to identify potential therapeutic targets of Sch A for TNBC.

### 2.2 Construction of Protein–Protein Interaction (PPI) Network

The intersection targets were uploaded to the STRING database (species: *Homo sapiens* ) with an interaction score of ≥ 0.7, indicating high confidence to acquire protein–protein interaction (PPI) data. Cytoscape version 3.9.1 was employed to construct the ‘Schisandrin A—Target—TNBC’ association network, wherein nodes represent compounds, targets and diseases, and edges denote interaction relationships. The Network Analyser tool within Cytoscape was utilized to compute the topological parameters, specifically the Degree value, of the targets; this Degree value indicates the connectivity strength of a target within the PPI network. A bar chart was generated based on the ranking of Degree values to identify high‐Degree core targets. Subsequently, the intersection targets were analysed using the R package clusterProfiler for Gene Ontology (GO) functional enrichment, encompassing biological process (BP), molecular function (MF) and cellular component (CC) categories, as well as KEGG pathway enrichment analysis. Enrichment results demonstrating statistical significance were identified using criteria of *p* < 0.05 and a false discovery rate (FDR) < 0.05, thereby elucidating the BPs and signalling pathways associated with the targets.

### 2.3 Construction of Prediction Model Using Random Forest Survival Algorithm

Gene expression and clinical follow‐up data, encompassing survival status and survival time, for patients with triple‐negative breast cancer (TNBC) were sourced from the METABRIC database. A preprocessing protocol was implemented to eliminate samples with missing data and to standardize the gene expression profiles. Concurrently, gene expression and clinical follow‐up data for TNBC patients from the independent test set GSE58812, obtained from the Gene Expression Omnibus (GEO) database, were acquired. These data underwent the same preprocessing procedures as the training set to facilitate subsequent validation of the model's generalization capability. In the analysis, potential therapeutic targets were considered, and univariate Cox proportional hazards regression analysis was employed to identify target genes associated with patient prognosis, using a significance threshold of *p* < 0.05. This process led to the identification of core target genes significantly linked to the prognosis of TNBC patients. Utilizing these core targets, a random forest prognostic model was developed with the R package randomForestSRC, specifying parameters of ntree = 100 and nodesize = 10. The model parameters were further refined through 5‐fold cross‐validation. Individual risk scores were derived from model predictions, and samples were categorized into high‐risk and low‐risk groups based on the median risk score. The Kaplan–Meier method was employed to estimate the survival curves of patients within these groups, and the Log‐rank test was utilized to assess survival differences between them. Furthermore, the model's generalization capability was validated using the test set GSE58812. The R package timeROC was applied to compute the area under the curve (AUC) values at various time intervals to evaluate the model's predictive performance.

### 2.4 Pan‐Cancer Gene Expression and Differential Analysis

Transcriptome data from pan‐cancer tumour tissues and their corresponding normal tissues were sourced from The Cancer Genome Atlas (TCGA) and the Genotype‐Tissue Expression (GTEx) databases, respectively. This dataset encompasses 33 types of malignant tumours from the TCGA database and matched normal tissue samples from the GTEx database. The data were normalized to the transcripts per million reads (TPM) format, and genes exhibiting low expression (defined as samples with TPM < 1 in more than 50% of cases) were excluded from the analysis. To assess the differential expression of core targets between tumour and normal tissues, the Wilcoxon rank‐sum test was employed, with a significance threshold set at *p* < 0.01. A pan‐cancer expression radar chart was subsequently generated using the R package ggplot2.

### 2.5 Immune Infiltration Analysis

Pan‐cancer expression data for core targets, along with corresponding immune cell infiltration data, were sourced from the TIMER 2.0 database. To assess the correlation between core target expression and immune cell infiltration, Spearman rank correlation analysis was employed. The relationship between targets and activated dendritic cells (aDC) was illustrated using scatter plots, while correlation heatmaps were utilized to depict the association characteristics between targets and various pan‐cancer immune cells. All analyses were conducted using the R software version 4.3.0, with the ‘corrplot’, ‘ggplot2’, and ‘pheatmap’ packages facilitating the visualization of results. A *p*‐value of less than 0.05 was considered indicative of statistical significance in the correlation analyses.

### 2.6 Molecular Docking Verification and Visualization

The two‐dimensional structure of the Sch A ligand was retrieved from the PubChem database (http://pubchem.ncbi.nlm.nih.gov/). This 2D structure was subsequently imported into ChemOffice 20.0 software to generate its three‐dimensional structure, which was then saved as a mol2 file. Concurrently, the RCSB PDB database (http://www.rcsb.org/) was utilized to identify crystal structures of core protein targets with a resolution of ≤ 2.5 Å. PyMOL 2.6.0 software facilitated preprocessing operations, including dehydration and dephosphorylation of the protein, which was subsequently saved as a PDB file. The Molecular Operating Environment (MOE) 2019 software was employed for energy minimization of the compound, preprocessing of the target protein and identification of the active pocket. To investigate the interactions between small molecule active components and protein targets, molecular docking was conducted using MOE 2019 software, with 50 docking runs specified. The binding activity between the ligand and protein was assessed based on binding energy, and the results were visualized using PyMOL 2.6.0 and Discovery Studio 2019 software.

### 2.7 Cell Culture

The MDA‐MB‐231 human breast cancer cell line was maintained in high‐glucose DMEM supplemented with 10% foetal bovine serum and sub‐cultured in a temperature‐controlled incubator set at 37°C with 5% CO_2_. Sch A, with a molecular weight of 416.507 g/mol, was dissolved in dimethyl sulfoxide (DMSO) to create a 100 mmol·L^−1^ stock solution. For experimental purposes, this stock solution was diluted with the culture medium to achieve the desired concentrations. The concentration gradient for proliferation assays specifically ranged at 0, 18, 27, 36, 54, 72, 108, 144, 216 and 288 μmol·L^−1^, while for apoptosis and cell cycle assays, the gradient was set at 0, 30 and 60 μmol·L^−1^. The final concentration of DMSO was maintained at ≤ 0.1% to ensure non‐cytotoxic conditions.

### 2.8 CCK‐8 Assay for Cell Proliferation

Cells in the logarithmic growth phase were seeded into 96‐well plates at a density of 5 × 10^3^ cells per well. Following cell adhesion, varying concentrations of Sch A were administered for a 48‐h treatment period. Each concentration was tested in quintuplicate, and the entire experiment was independently replicated three times. Subsequently, 10 μL of CCK‐8 reagent was added to each well, and the plates were incubated for an additional 2 h. The optical density at 450 nm (OD₄₅₀ₙₘ) was then measured using a Thermo Multiskan FC microplate reader.

### 2.9 Annexin V‐PI Double Staining for Cell Apoptosis

Following a 48‐h treatment of cells with varying concentrations of Sch A, 1 × 10^5^ cells were harvested and subjected to two washes with pre‐cooled PBS. In accordance with the protocol provided by the Annexin V‐FITC Cell Apoptosis Detection Kit, the cells were resuspended in 500 μL of Binding Buffer. Subsequently, 5 μL of Annexin V‐FITC and 5 μL of PI staining solution were added, and the mixture was incubated in the dark for 15 min. Cell apoptosis was assessed using a flow cytometer (BD FACS Canto II), and the data were analysed with Flow Jo V10 software.

### 2.10 PI Single Staining for Cell Cycle Analysis

Following a 48‐h treatment of cells with varying concentrations of Sch A, a total of 1 × 10^5^ cells were harvested and subsequently fixed in 70% pre‐cooled ethanol at 4°C overnight. Post‐fixation, the cells were washed with PBS, and 100 μL of RNase A (100 μg·mL^−1^) was added, followed by incubation at 37°C for 30 min. Subsequently, 400 μL of propidium iodide (PI) staining solution (50 μg·mL^−1^) was introduced, and the mixture was incubated in the dark for an additional 30 min. Cell cycle distribution was assessed using a BD FACS Canto II flow cytometer, and data analysis was conducted with Flow Jo V10 software to determine the proportions of cells in the G₀/G₁, S and G_2_/M phases.

### 2.11 Transcriptome Sequencing and Bioinformatics Analysis

MDA‐MB‐231 cells from both the control group and the Sch A (30 μM)‐treated group, each comprising two biological replicates, were harvested for analysis. Total RNA was extracted utilizing the TRIzol reagent. Paired‐end sequencing (PE150) was conducted on the Illumina NovaSeq 6000 platform. Differentially expressed genes (DEGs) were identified using DESeq2 software, applying criteria of |log_2_ (Fold Change) | ≥ 1 and *p* < 0.05. The results were visualized through the generation of volcano plots and cluster heatmaps. The R package clusterProfiler was employed for Gene Ontology (GO) functional enrichment and Kyoto Encyclopedia of Genes and Genomes (KEGG) pathway enrichment analyses, utilizing significance thresholds of *p* < 0.05 and a false discovery rate (FDR) < 0.05. Visual representations of the results were generated in the form of bubble plots and bar charts. Additionally, pathway enrichment trends were assessed using Gene Set Enrichment Analysis (GSEA) software, and GSEA plots were produced to elucidate the expression changes in key pathways modulated by Sch A. The raw sequencing data obtained in this study have been deposited in the Gene Expression Omnibus (GEO) database under accession number GSE28358, thereby ensuring data accessibility and reproducibility in accordance with open science principles.

### 2.12 Statistical Analysis

Statistical analyses in this study were conducted utilizing R software version 4.3.0 and GraphPad Prism software version 9.0. Data from cell experiments are presented as mean ± standard deviation (SD). A one‐way analysis of variance (ANOVA) was employed to assess differences among multiple groups, followed by Tukey's post hoc test for pairwise comparisons. A *p*‐value of less than 0.05* was deemed statistically significant, while a *p*‐value of less than 0.01** was considered highly statistically significant.

## 3 Results

### 3.1 Screening of Potential Targets of Sch A and TNBC‐Related Targets

Figure 1A illustrates the three‐dimensional (3D) structure of Sch A. Potential targets of Sch A were identified through comprehensive searches across several databases, including SEA, SWISS Target Prediction and Super‐PRED. After eliminating duplicate entries and applying a confidence score threshold of greater than 0.7, a total of 178 potential targets for Sch A were identified. Targets related to triple‐negative breast cancer (TNBC) were compiled from the GeneCards and OMIM databases, resulting in 6856 unique TNBC‐related targets after deduplication. The intersection of Sch A's potential targets and TNBC‐related targets was analysed using Venny 2.1, revealing 128 common targets, which were subsequently designated as potential anti‐TNBC targets of Sch A (Figure 1B,C).

### 3.2 Identification of Core Targets

The 128 identified common targets were subsequently imported into the STRING database to construct a protein–protein interaction (PPI) network. Additionally, the overlapping targets were uploaded into Cytoscape 3.8 software, where a core target network was developed through topological analysis (Figure 1D). By sorting the targets in descending order based on their Degree values, 36 core targets were identified using a cutoff value of Degree > 15. These targets include SRC, EGFR, HIF1A, PTGS2, NFKB1, ERBB2, GSK3B, TLR4, PIK3CA, CREBBP, KDR, BRAF, AR, ABCB1, BRD4, NFE2L2, KIT, CYP2C19, SLC2A1, CYP2C9, ABCG2, TGFBR1, KEAP1, CDK1, MET, DRD2, IDO1, TNFRSF1A, IKBKB, ALK, TOP2A, MAOA, PTGS1, FLT1, PKM and NOS3. The analytical results suggest that these targets may act as key mediators in the therapeutic effects of Sch A on triple‐negative breast cancer (TNBC) (Figure 1E).

### 3.3 Functional Enrichment Analysis of Common Targets

**FIGURE 2:**
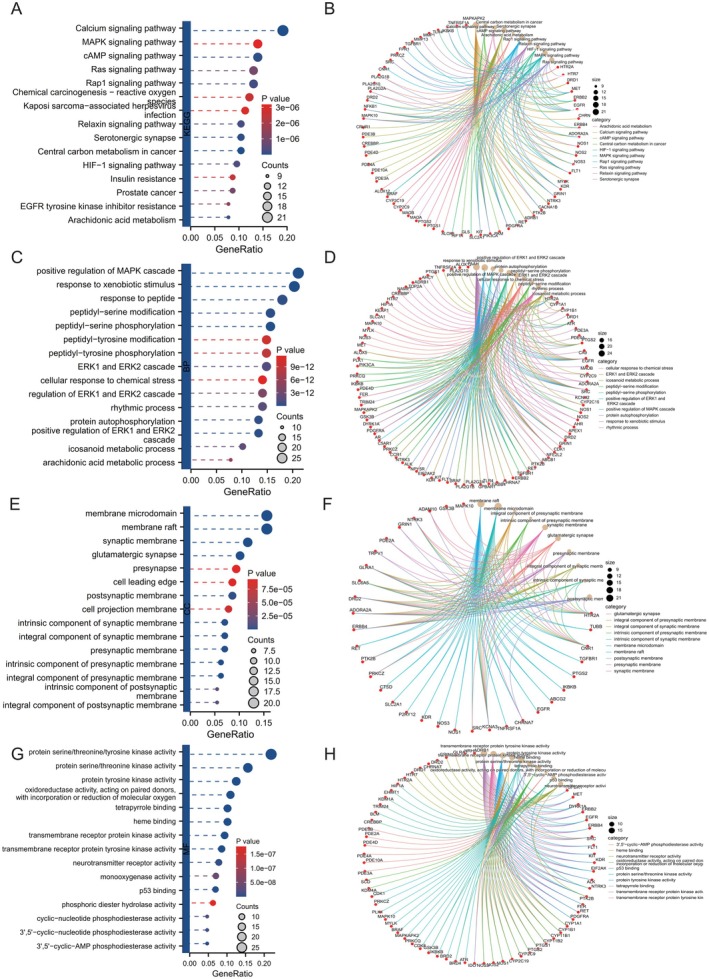
KEGG and GO enrichment analysis diagram of the intersectional targets of schisandrin A and TNBC. The top 15 significantly enriched pathways are displayed. The y‐axis represents the pathway name, and the x‐axis represents the enrichment score. The colour gradient indicates the *p‐*value, with darker colours representing higher statistical significance. (A, B) Diagram illustrating KEGG pathway enrichment analysis; (C–H) Diagram depicting GO enrichment analysis, with (C, D) representing biological processes (BP), (E, F) representing molecular functions (MF) and (G, H) representing cellular components (CC).

Pathway enrichment analyses using the Kyoto Encyclopedia of Genes and Genomes (KEGG) and Gene Ontology (GO) were conducted on the 128 common targets via the ClusterProfiler package. The KEGG pathway enrichment analysis identified 130 pathways with significant enrichment (*p* < 0.05). Among these, the top 15 enriched pathways were the ‘MAPK signaling pathway’, ‘Ras signaling pathway’, ‘HIF‐1 signaling pathway’, ‘EGFR tyrosine kinase inhibitor resistance’ and ‘Calcium signaling pathway’ (refer to Figure 2A,B). Concurrently, the GO enrichment analysis revealed 1792 significantly enriched terms (*p* < 0.05), comprising 1540 BP terms, 80 cellular component (CC) terms and 172 molecular function (MF) terms. The top 15 enriched BP terms included ‘positive regulation of MAPK cascade’, ‘cellular response to chemical stress’ and ‘ERK1 and ERK2 cascade’ (refer to Figure 2C,D). The most significantly enriched cellular component (CC) terms identified were ‘membrane microdomain’, ‘membrane raft’ and ‘cell leading edge’ (refer to Figure 2E,F). The major molecular function (MF) terms included ‘protein kinase activity’, ‘transmembrane receptor protein kinase activity’ and ‘p53 binding’ (refer to Figure 2G,H). These pathways are intricately linked to tumour proliferation, apoptosis, angiogenesis and immune regulation, indicating that Sch A may exert anti‐triple‐negative breast cancer effects through the modulation of these signalling pathways.

### 3.4 Screening of Prognostic Core Targets and Construction of Prognostic Model

**FIGURE 3:**
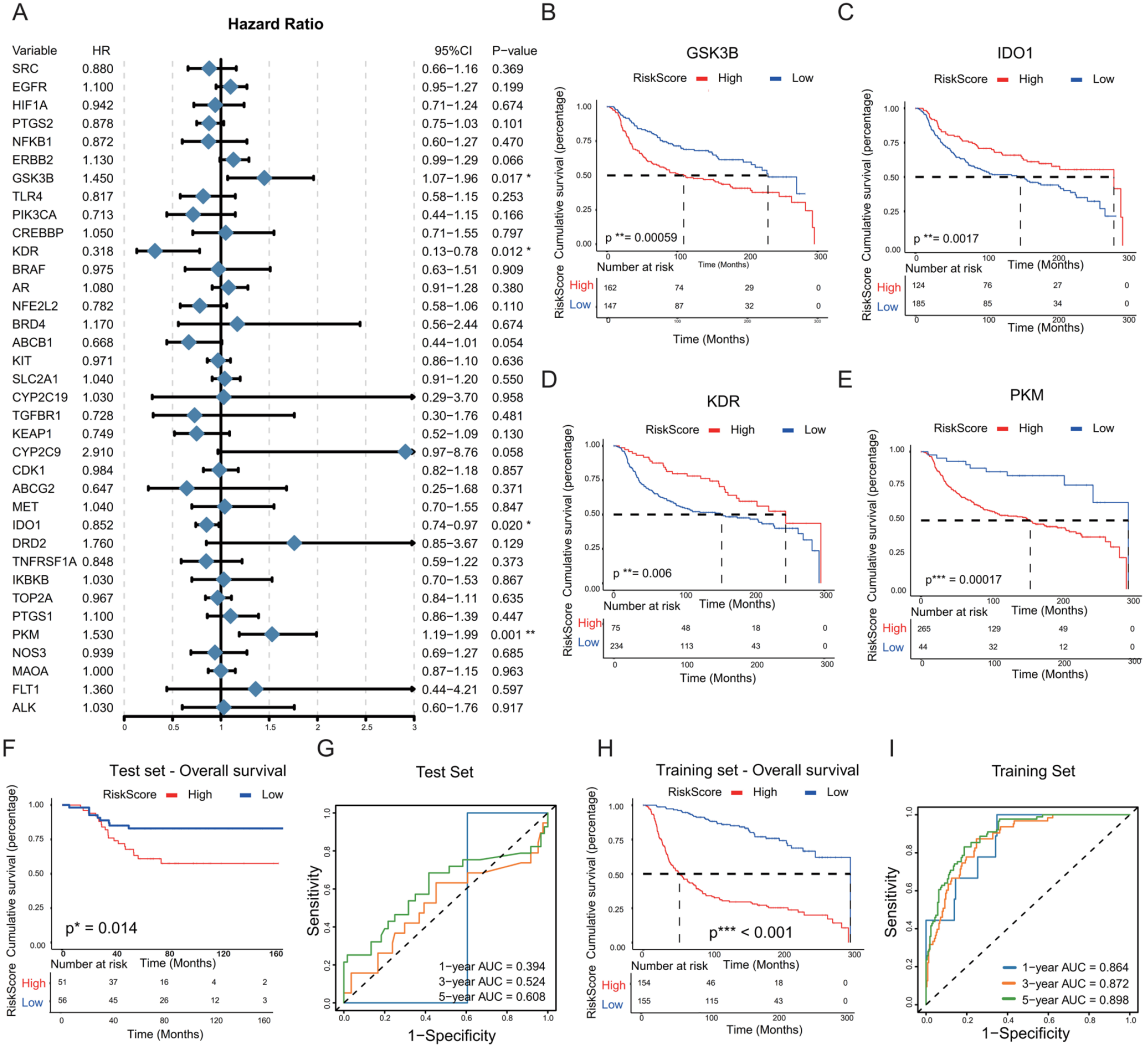
Identification of prognostic core targets and evaluation of the constructed risk prediction model. (A) Forest plot of univariate Cox regression analysis for putative target genes. (B–E) Kaplan–Meier overall survival curves based on the expression of (B) GSK3B, (C) IDO1, (D) KDR and (E) PKM. (F, G) Kaplan–Meier survival curve (F) and time‐dependent ROC curves for 1‐, 3‐ and 5‐year overall survival (G) of the risk model in the test cohort. (H, I) Corresponding Kaplan–Meier survival curve (H) and time‐dependent ROC curves (I) in the training cohort (*p* < 0.05, **p < 0.01, ***p < 0.001).

To further prioritize these candidate targets with clinical prognostic significance, the 36 core targets were subjected to analysis using the random forest survival algorithm, leveraging data from the METABRIC database. This analysis identified four core targets—GSK3B, IDO1, KDR and PKM—that are closely associated with overall survival (OS) in patients with triple‐negative breast cancer (Figure 3A). Notably, high expression levels of GSK3B were found to be negatively correlated with the prognosis of TNBC patients (*p* = 0.00059, Figure 3B), whereas increased expression of IDO1 demonstrated a positive correlation with patient outcomes (*p* = 0.0017, Figure 3C). Furthermore, patients exhibiting high KDR expression experienced favourable prognostic outcomes (*p* = 0.006, Figure 3D), while elevated PKM expression was negatively associated with TNBC prognosis (*p* = 0.00017, Figure 3E). A prognostic model was developed utilizing four specific targets, enabling the stratification of patients in the training cohort into high‐risk and low‐risk categories based on the median risk score. The model's generalization capability was assessed using the GSE58812 dataset as an independent validation set. The findings indicated a negative correlation between high expression levels of the core targets and the prognosis of TNBC patients in both the model and training cohorts (*p* < 0.05, Figures 3F and 3H). Analysis of the receiver operating characteristic (ROC) curve demonstrated that the model exhibited strong accuracy and discriminative power in predicting overall survival (OS) within the training cohort, yielding AUC values of 0.864, 0.872 and 0.898 at 1, 3 and 5 years, respectively (Figure 3I). In contrast, the model cohort exhibited attenuated AUC values of 0.394, 0.524 and 0.608 (Figure 3G). This discrepancy highlights the profound inter‐tumoral and intra‐tumoral heterogeneity inherently characteristic of TNBC. Furthermore, the lower predictive performance in the validation set is likely attributable to disparities in clinical baseline characteristics across diverse patient populations, inherent data heterogeneity between sequencing platforms (METABRIC vs. GEO) and potential overfitting within the training phase due to sample size constraints.

### 3.5 Expression Profiles of the Four Core Targets in Pan‐Cancer Cohorts

**FIGURE 4:**
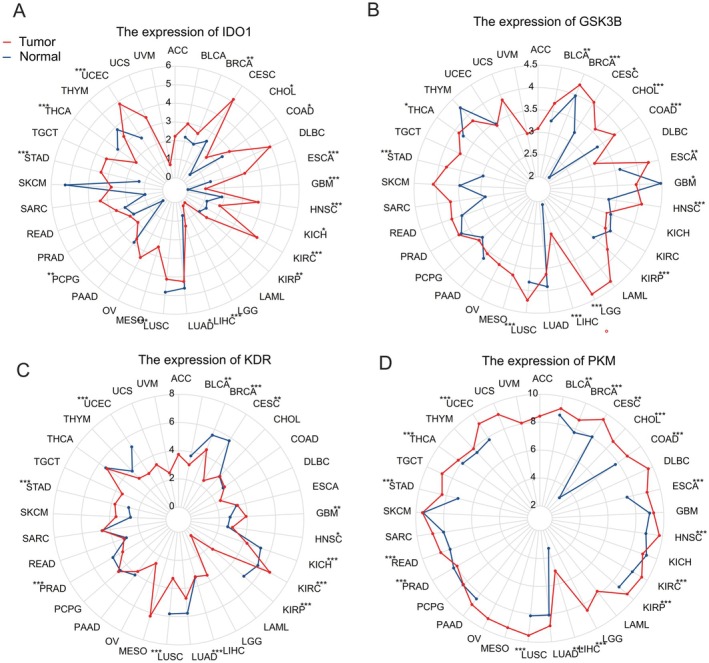
A radar chart illustrating the expression differences of GSK3B, IDO1, KDR and PKM within a pan‐cancer cohort is presented. This chart delineates the differential expression of these four core targets—IDO1 (A), GSK3B (B), KDR (C) and PKM (D)—comparing tumour tissues (depicted by the red curve) to normal tissues (depicted by the blue curve) across the pan‐cancer cohort. The circumferential axis denotes various cancer types (e.g., UCS, UVM, ACC), while the radial axis indicates gene expression levels (*p* < 0.05, **p < 0.01, ***p < 0.001).

Subsequent analyses utilized radar plots to assess the differential expression of four core targets—IDO1, GSK3B, KDR and PKM—between tumour tissues (represented by red curves) and normal tissues (represented by blue curves) within a pan‐cancer cohort. The expression of IDO1 was found to be significantly elevated in breast invasive carcinoma (BRCA), cholangiocarcinoma (CHOL), colon adenocarcinoma (COAD) and oesophageal carcinoma (ESCA), whereas it was notably reduced in lung squamous cell carcinoma (LUSC) and thyroid carcinoma (THCA) (*p* < 0.01, as depicted in Figure 4A). GSK3B expression was upregulated in bladder urothelial carcinoma (BLCA), BRCA, endocervical adenocarcinoma (CESC) and CHOL, while it was downregulated in glioblastoma multiforme (GBM) (*p* < 0.01, as shown in Figure 4B). KDR expression was significantly diminished in tumour tissues of BLCA, BRCA, CESC and uterine corpus endometrial carcinoma (UCEC), yet it was elevated in GBM and kidney renal clear cell carcinoma (KIRC) compared to normal tissues (*p* < 0.01, illustrated in Figure 4C). PKM exhibited a trend of pan‐cancer upregulation in tumours, with markedly higher expression levels in BLCA, BRCA, CESC, CHOL and lung adenocarcinoma (LUAD) (*p* < 0.01, as presented in Figure 4D). Collectively, the widespread overexpression of GSK3B, IDO1, KDR and PKM in tumour tissues across various cancers suggests their potential role as oncogenic drivers.

### 3.6 Analysis of Correlation Between Prognostic Core Targets and Immune Infiltration

**FIGURE 5:**
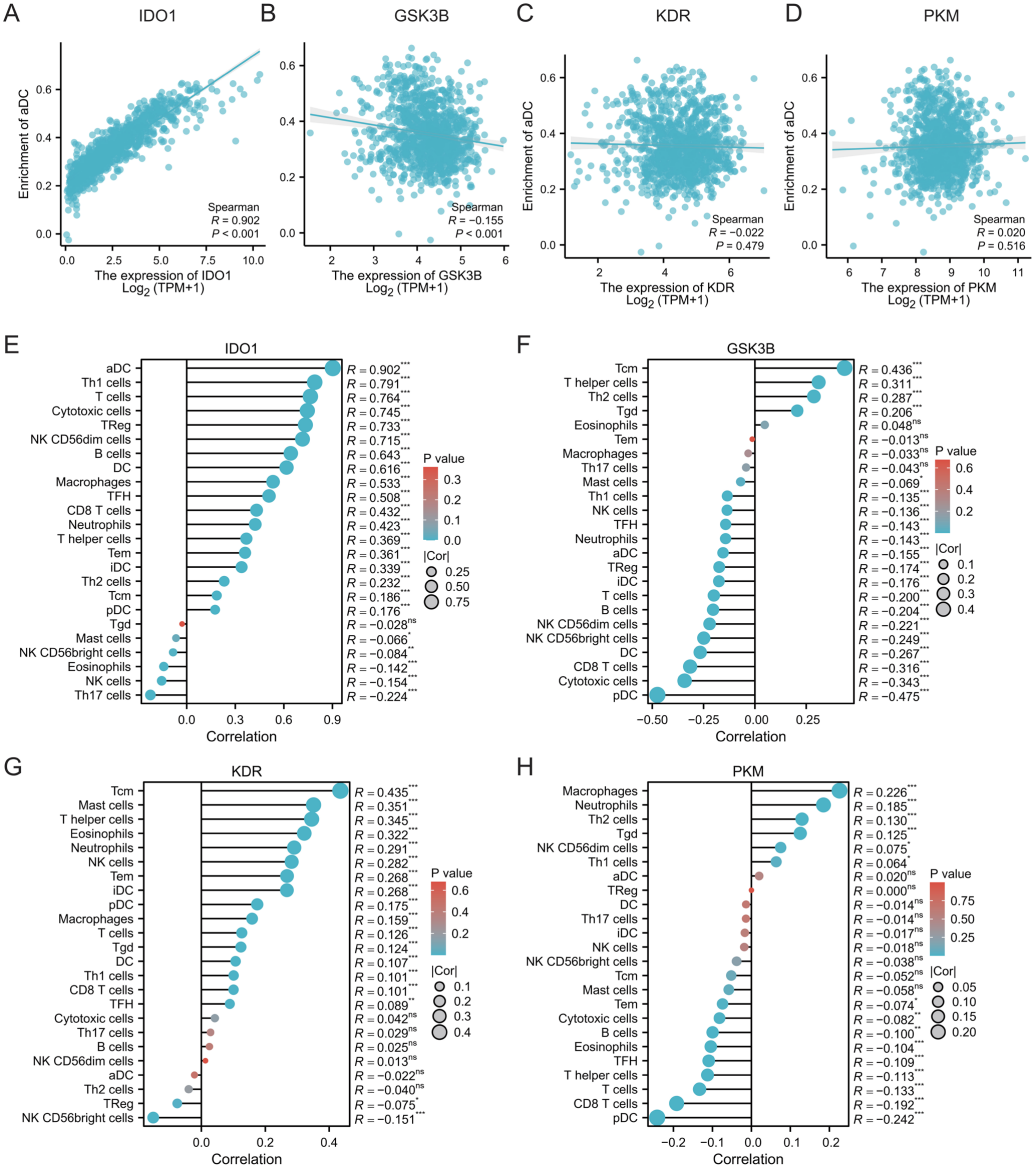
Correlation between 4 prognostic core targets and immune cell infiltration in TNBC. (A–D) Scatter plots showing the correlation between IDO1/GSK3B/KDR/PKM expression and activated dendritic cell (aDC) infiltration. (E–H) Heatmap depicting the Spearman correlation coefficients between core targets (IDO1, GSK3B, KDR, PKM) and various immune cell subsets. Red indicates positive correlation, blue indicates negative correlation and the intensity of the colour represents the correlation strength (*p* < 0.05, **p < 0.01, ***p < 0.001).

The correlation between the four prognostic core targets (IDO1, GSK3B, KDR, PKM) and the enrichment level of APCs (aDCs) in TNBC was analysed using the Spearman correlation analysis. The findings indicated a strong positive correlation between IDO1 expression and aDCs (*R* = 0.902, *p* < 0.001, Figure 5A). Conversely, GSK3B expression demonstrated a significant negative correlation with aDC enrichment (*R* = −0.155, *p* < 0.001, Figure 5B). In contrast, neither KDR (*R* = 0.022, *p* = 0.479, Figure 5C) nor PKM (*R* = 0.020, *p* = 0.516, Figure 5D) expression exhibited a significant correlation with aDC enrichment.

Correlation heatmaps were employed to examine the relationships between gene expression and the infiltration of various immune cell types across pan‐cancer cohorts. The expression of IDO1 demonstrated a significant positive correlation with the majority of immune cells, with the strongest association observed with aDCs (*R* = 0.902, *p* < 0.001) (Figure 5E). Additionally, IDO1 exhibited a high positive correlation with T helper 1 (Th1) cells (*R* = 0.791, *p* < 0.001) and regulatory T cells (Tregs) (*R* = 0.764, *p* < 0.001) (Figure 5F). In contrast, KDR expression showed a significant positive correlation with central memory T cells (Tcm) (*R* = 0.435, *p* < 0.001) and a significant negative correlation with natural killer (NK) CD56 bright cells (R = −0.151, *p* < 0.001), while displaying weak associations with other immune cell types (Figure 5G). PKM was found to have weak correlations with most immune cell subsets overall (Figure 5H). Overall, the expressions of IDO1, GSK3B, KDR and PKM exhibited distinct associations with components of the immune microenvironment, such as aDCs and specific immune cells, indicating that IDO1 and GSK3B may play roles in remodelling the tumour immune microenvironment through the regulation of immune cell infiltration.

### 3.7 Molecular Docking Verification

**FIGURE 6:**
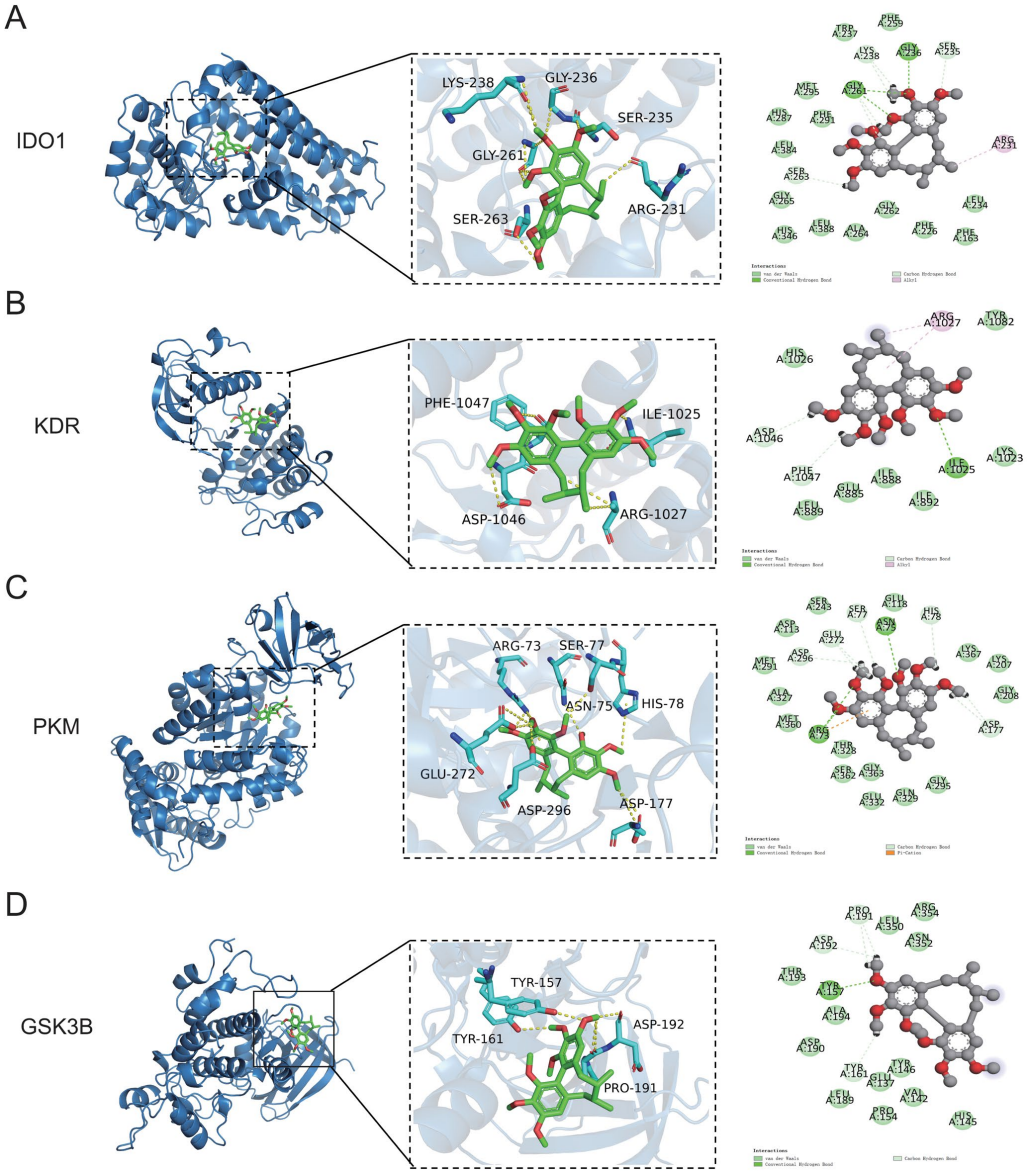
Molecular docking results of Sch A with 4 prognostic core targets. (A–D) Three‐dimensional binding modes of Sch A with IDO1 (A), KDR (B) and PKM (C), GSK3B (D), respectively. The green structure represents Sch A, and the grey structure represents the target protein. The dotted lines indicate hydrogen bonds between Sch A and the target protein. The binding energies are shown in the corresponding figures.

Molecular docking analyses were conducted to assess the binding affinity between Sch A and four prognostic core targets. The 3D structures of Sch A and the target proteins (GSK3B, IDO1, KDR, PKM) were obtained from the PubChem and Protein Data Bank (PDB) databases, respectively. The docking results indicated that Sch A exhibited stable binding interactions with all four core targets. The binding energies for Sch A with IDO1, GSK3B, KDR and PKM were calculated to be −6.6433 kcal/mol (Figure 6A), −5.8660 kcal/mol (Figure 6D), −5.8562 kcal/mol (Figure 6B) and −6.1404 kcal/mol (Figure 6C), respectively. A binding energy of less than −5 kcal/mol is indicative of a stable interaction, suggesting that Sch A may exert anti‐TNBC effects through direct binding to these core targets.

### 3.8 In Vitro Experimental Validation

**FIGURE 7:**
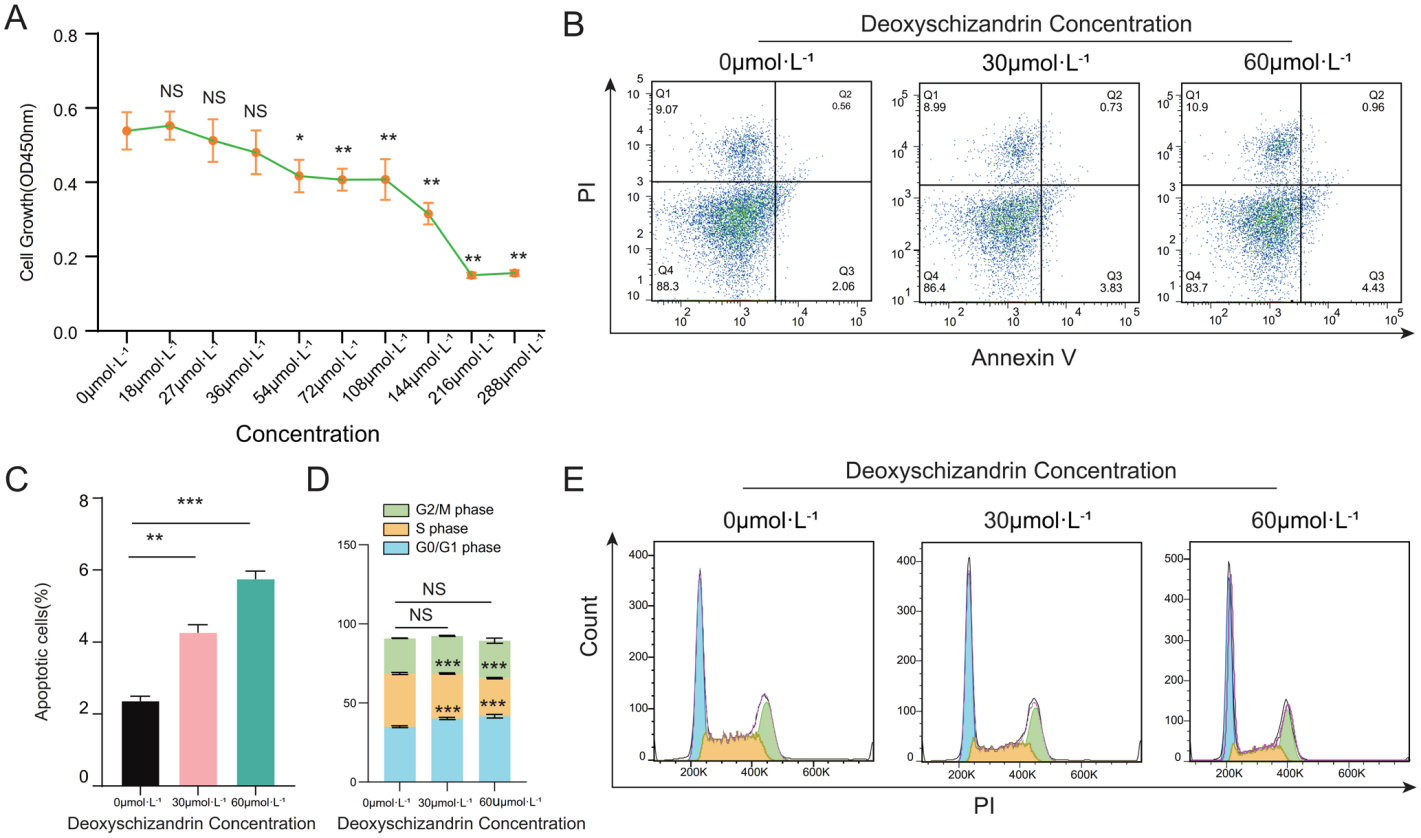
In vitro effects of Sch A on MDA‐MB‐231 cell proliferation, apoptosis and cell cycle. (A) CCK‐8 assay showing the concentration‐dependent inhibitory effect of Sch A on cell proliferation. (B) Flow cytometry analysis of cell apoptosis after Sch A treatment. (C) The histogram shows the statistical analysis of apoptosis rates. (D) Cell cycle distribution analysed by PI staining. (E) The histogram shows the proportion of cells in G_0_/G_1_, S and G_2_/M phases (*p* < 0.05, **p < 0.01, ***p < 0.001).

To validate the anti‐tumour effects of Sch A in vitro, we assessed its impacts on the proliferation, apoptosis and cell cycle of MDA‐MB‐231 cells through a series of experiments. The CCK‐8 assay was utilized to quantify the impact of Sch A on the proliferative capacity of MDA‐MB‐231 cells. The findings revealed no statistically significant alteration in cell proliferation at Sch A concentrations ≤ 36 μmol·L^−1^ (*p* > 0.05). However, at concentrations ≥ 54 μmol·L^−1^, a marked inhibition of cell proliferation was observed in a concentration‐dependent manner (*p* < 0.05) (Figure 7A). To assess the apoptotic effects of Sch A, Annexin V‐PI double‐staining flow cytometry was employed. Relative to the 0 μmol·L^−1^ control group, the apoptosis rate of MDA‐MB‐231 cells was significantly elevated following exposure to 30 μmol·L^−1^ Sch A (*p* < 0.01), with a further increase observed at 60 μmol·L^−1^ (*p* < 0.001) (Figure 7B,C), suggesting that Sch A induces apoptosis in a concentration‐dependent fashion. Additionally, cell cycle analysis revealed that Sch A specifically induced arrest of MDA‐MB‐231 cells at the S phase (Figure 7D,E). The percentage of cells in the S phase was significantly elevated in the Sch A‐treated groups compared to the control group (*p* < 0.05). Overall, Sch A demonstrates inhibitory effects on the growth of MDA‐MB‐231 cells by suppressing proliferation, inducing apoptosis and causing cell cycle arrest at the S phase.

### 3.9 Transcriptomic Expression and Functional Enrichment Characteristics of MDA‐MB‐231 Cells Following Schisandrin A Treatment

**FIGURE 8:**
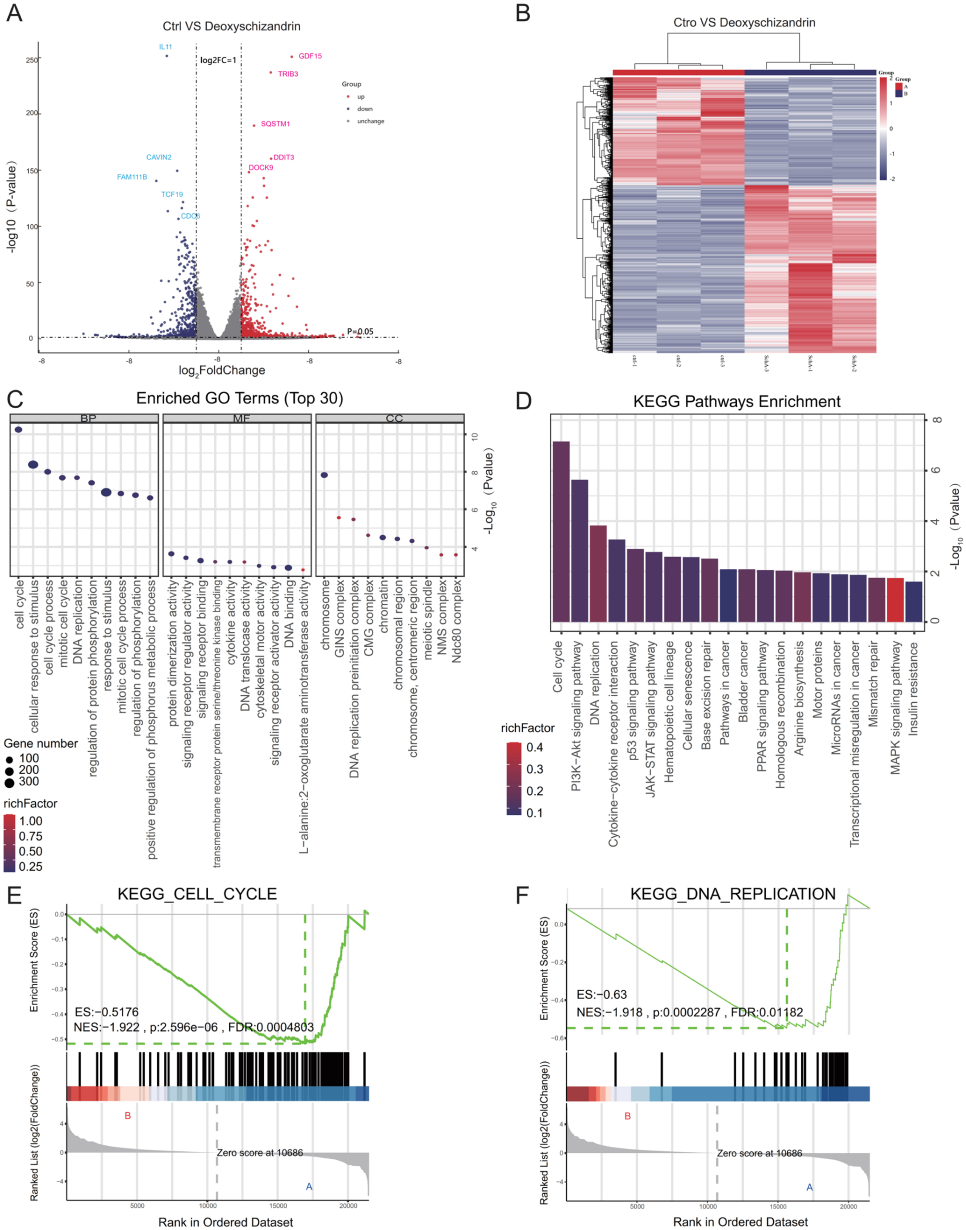
Transcriptome analysis of Sch A‐treated MDA‐MB‐231 cells. (A, B) Volcano plot and heat map of differentially expressed genes (DEGs). Red dots represent upregulated genes, blue dots represent downregulated genes and black dots represent non‐differentially expressed genes (|log_2_ (Fold Change)| ≥ 1, *p* < 0.05). (C, D) KEGG pathway enrichment bubble plots (C) and GO functional enrichment bar charts (D) of DEGs. The size of the bubbles indicates the number of genes, and the colour represents the *p‐*value. (E, F) GSEA plot for the cell cycle pathway (KEGG_CELL_CYCLE, KEGG_DNA_REPLICATION). The standardized enrichment score (NES), *p*‐value and false discovery rate (FDR) are annotated (*p* < 0.05, **p < 0.01, ***p < 0.001).

Considering that Sch A inhibits proliferation, induces apoptosis and arrests the cell cycle in MDA‐MB‐231 cells, as previously demonstrated, transcriptome sequencing was performed to analyse the gene expression profile of MDA‐MB‐231 cells treated with Sch A. A total of 5082 differentially expressed genes (DEGs) were identified, comprising 625 upregulated genes and 4457 downregulated genes (see Figure 8A,B). Gene Ontology (GO) and Kyoto Encyclopedia of Genes and Genomes (KEGG) enrichment analyses of the DEGs revealed that the downregulated genes were predominantly associated with the ‘cell cycle’, ‘DNA replication’, ‘MAPK signaling pathway’ and ‘PI3K‐Akt signaling pathway’ (refer to Figure 8C,D), findings that align with the results of the network pharmacology analysis. Notably, the ‘MAPK signaling pathway’ demonstrated consistency with the network pharmacology outcomes. The ‘cell cycle’ pathway exhibited the highest significance in terms of enrichment and enrichment factor, indicating that it is the primary pathway through which Sch A modulates cell proliferation (see Figure 8D). Subsequently, GSEA provided further confirmation that the ‘cell cycle’ pathway (NES = −1.922, *p* < 0.001) (Figure 8E) and the ‘DNA replication’ pathway (NES = −1.918, *p* < 0.001) (Figure 8F) were significantly downregulated following treatment with Sch A. Collectively, the differentially expressed genes (DEGs) in MDA‐MB‐231 cells treated with Sch A are primarily associated with BPs and pathways such as cell cycle regulation and signal transduction. These findings offer transcriptional‐level experimental evidence that contributes to the elucidation of the molecular mechanisms underlying the effects of Sch A on breast cancer cells.

## 4 Discussion

Triple‐negative breast cancer is characterized by its complex molecular heterogeneity and the absence of specific therapeutic targets, resulting in a limited availability of targeted treatments and an exceedingly poor prognosis. Consequently, the investigation of novel and effective therapeutic strategies has emerged as a primary focus within oncological research [3, 4]. Natural products have attracted considerable attention in the fields of cancer research and drug development due to their distinctive ability to exert multi‐target and multi‐pathway regulatory effects [5]. SchA, a principal active constituent of Schisandra chinensis, has been demonstrated to exhibit a range of pharmacological activities, including anti‐inflammatory, antioxidant and anti‐tumour effects [17, 18, 19, 20]. Nevertheless, the regulatory function of SchA in TNBC and the associated mechanisms are not yet fully understood. This study provides a comprehensive elucidation of the primary targets and potential mechanisms of SchA in combating TNBC by employing an integrated methodological approach that combines network pharmacology, experimental validation and transcriptome sequencing.

Through the integration of target information from multiple databases, we identified 128 intersecting targets between SchA and triple‐negative breast cancer. Subsequent topological analysis refined this list to 36 core targets. Utilizing a Random Forest survival algorithm in conjunction with the METABRIC clinical database, we further identified four core targets—GSK3B, IDO1, KDR and PKM—that exhibit a significant association with TNBC patient prognosis. The prognostic model demonstrated strong predictive performance when validated against the independent test set GSE58812. These findings indicate that these four targets are not only pivotal molecular nodes in SchA's mechanism of action against TNBC but also hold promise as biomarkers for assessing TNBC prognosis. Glycogen synthase kinase‐3β (GSK3β) is critically involved in the pathogenesis of various cancer types, including bladder, pancreatic and lung cancers. It modulates tumour cell proliferation, apoptosis, invasion, metastasis and the immune microenvironment through the regulation of key signalling pathways such as Wnt/β‐catenin, PI3K/AKT and NF‐κB [24, 25]. In the present study, GSK3B was identified as a potential core target for the action of SchA against triple‐negative breast cancer. Consistent with previous studies, elevated expression of GSK3B was significantly correlated with poor prognosis in TNBC patients. Molecular docking analysis revealed a binding energy of −5.8660 kcal/mol between SchA and GSK3B, suggesting a potentially stable interaction. These findings imply that SchA may exert its anti‐TNBC effects by targeting and inhibiting GSK3B activity.

Indoleamine 2,3‐dioxygenase 1 (IDO1), a critical rate‐limiting enzyme within the tryptophan‐kynurenine metabolic pathway, is instrumental in facilitating tumour immune evasion. Our investigation, utilising network pharmacology and survival analysis, identified IDO1 as a potential primary target for SchA in the treatment of triple‐negative breast cancer. Elevated expression levels of IDO1 were correlated with improved prognostic outcomes in TNBC patients (*p* = 0.0017), consistent with certain extant studies [26]. IDO1 contributes to the establishment of an immunosuppressive tumour microenvironment by catalysing the degradation of tryptophan, leading to local tryptophan depletion and the accumulation of metabolites such as kynurenine. This biochemical process impairs T cell functionality and facilitates the differentiation of regulatory T cells (Tregs), thereby enabling tumour cells to evade immune detection and destruction [27, 28]. In this study, further analysis of immune infiltration revealed a strong and positive correlation between IDO1 expression and the enrichment level of APCs (aDCs) (*R* = 0.902, *p* < 0.001). Additionally, IDO1 expression was closely associated with immune cells such as Th1 cells and Tregs, highlighting its pivotal role in modulating the immune microenvironment of triple‐negative breast cancer. Molecular docking studies demonstrated a high binding affinity between SchA and IDO1, with a binding energy of −6.6433 kcal/mol. Given this strong affinity, we postulate that SchA may effectively occupy the active catalytic pocket of IDO1. By acting as a potential competitive inhibitor, SchA could directly suppress the specific enzymatic activity of IDO1, primarily its role in tryptophan catabolism. Theoretically, this targeted blockade could inhibit the generation of immunosuppressive kynurenine metabolites, thereby potentially reversing T cell anergy and influencing the tumour immune microenvironment. However, it is imperative to acknowledge that our current findings are predominantly based on in silico predictions and lack direct in vivo experimental validation of immune regulation. Consequently, further in‐depth investigation is warranted to elucidate the specific in vivo role and immunomodulatory mechanism of SchA via IDO1 in TNBC.

KDR (VEGFR2), a pivotal regulator of angiogenesis, demonstrates variable expression patterns across different cancer types. In triple‐negative breast cancer tissues, its expression is reduced compared to normal tissues, and elevated levels of KDR are associated with improved prognostic outcomes. This phenomenon may be attributed to KDR's dual functionality in modulating both tumour angiogenesis and the recruitment of immune cells [29, 30]. Conversely, PKM, a critical enzyme in glycolysis, is overexpressed in numerous tumours, including TNBC, and its elevated expression is inversely related to patient prognosis. This observation underscores the reliance of TNBC cells on aerobic glycolysis, commonly referred to as the Warburg effect [31]. Molecular docking studies have identified a notably low binding energy between SchA and PKM (−6.1404 kcal/mol). Future empirical validations involving specific metabolic flux assays are required to definitively confirm SchA's regulatory impact on PKM‐mediated glycolysis. Collectively, these four core targets address multiple cancer hallmarks, such as proliferation, metabolism, angiogenesis and immune regulation, thereby constituting the central molecular target network through which SchA mediates its anti‐TNBC effects. Furthermore, the pan‐cancer abnormal expression of core targets (GSK3B, IDO1, KDR and PKM) strongly suggests that the anti‐tumour potential of Schisandrin A may not be limited to TNBC. Exploring its therapeutic efficacy in other malignancies represents a promising direction for our future research.

Cellular functional assays have demonstrated that SchA inhibits the proliferation of MDA‐MB‐231 cells in a concentration‐dependent manner, induces apoptosis and specifically arrests the cell cycle at the S phase. These findings align with previous reports of SchA's inhibitory effects on lung cancer cell proliferation [18]. However, this study is the first to delineate the specific phase of cell cycle arrest (S phase) and the apoptosis‐inducing effect in triple‐negative breast cancer cells. Arresting the cell cycle in the S phase is critical for DNA replication, as it can directly impede tumour cell DNA synthesis and subsequent proliferation [32]. In this study, SchA treatment resulted in a significant reduction in the proportion of cells in the G0/G1 phase, accompanied by an increase in the proportion of cells in the S phase. Transcriptomic analysis further identified a marked downregulation of the ‘Cell cycle’ and ‘DNA replication’ pathways. These findings suggest that SchA may inhibit the expression of proteins related to DNA replication, thereby obstructing S phase progression and ultimately suppressing cell proliferation. This evidence provides direct experimental support for SchA's functional mechanism against TNBC. Transcriptome sequencing and functional enrichment analysis demonstrated a significant alteration in the transcriptomic profile of MDA‐MB‐231 cells following SchA treatment. DEGs were predominantly enriched in categories such as ‘Cell cycle’, ‘DNA replication’ and ‘PI3K‐Akt signaling pathway.’ Notably, the ‘Cell cycle’ pathway exhibited the highest degree of enrichment significance and factor. GSEA corroborated a significant downregulation trend for this pathway, which is consistent with the experimental results indicating cell cycle arrest.

In conclusion, this study systematically elucidated the principal targets, key pathways and functional mechanisms underlying the anti‐triple‐negative breast cancer (TNBC) effects of Schisandrin A (SchA). The findings confirm that SchA exerts its anti‐tumour effects through the synergistic and multi‐target regulation of the cell cycle pathway. These results provide a theoretical foundation for the development of SchA as a potential therapeutic agent for TNBC and propose a comprehensive ‘multi‐technique integration’ strategy for investigating the anti‐tumour mechanisms of natural products.

However, this study is subject to certain limitations. The cell experiments were conducted using only a single TNBC cell line (MDA‐MB‐231), which lacks validation across other TNBC subtypes and may not fully represent the heterogeneity of TNBC. To address this, our subsequent research will incorporate a broader panel of TNBC cell lines (such as MDA‐MB‐468 and BT‐549) to comprehensively validate SchA's multi‐subtype efficacy. In vivo animal experiments were not performed to validate the efficacy and safety of SchA against triple‐negative breast cancer (TNBC); consequently, its mechanism of action in vivo necessitates further investigation. Moving forward, we plan to establish both human xenograft and immunocompetent syngeneic mouse models. These in vivo platforms will be pivotal for evaluating the pharmacokinetics, systemic biosafety and dynamic tumour‐suppressive capabilities of SchA. Network pharmacology predictions possess inherent limitations, and the interactions and regulatory relationships among the core targets require additional experimental validation through techniques such as co‐immunoprecipitation (Co‐IP) and Western blotting. Accordingly, our ongoing experimental pipeline explicitly includes systematic Western blotting to quantify the protein expression levels of the core targets (GSK3B, IDO1, KDR and PKM), as well as Co‐IP assays to physically verify the direct molecular interactions predicted by our docking models. Furthermore, the study did not thoroughly examine the specific regulatory effects of SchA on the tumour immune microenvironment and cell cycle signalling pathways, which warrant exploration in future research endeavours.

## Author Contributions

**Hongning Cai:** writing – review and editing, visualization, project administration, resources, supervision. **Yingchun Zhang:** methodology, data curation, validation, project administration. **Jianfang Guo:** conceptualization, funding acquisition, writing – review and editing, visualization, resources, supervision, project administration. **Meng Xu:** conceptualization, methodology, software, data curation, validation, writing – original draft. **Rubing Mei:** methodology, software, data curation, formal analysis, validation. **Tingting Xue:** methodology, validation, software, formal analysis, data curation. **Yu Zhou:** methodology, investigation, validation, software, formal analysis, data curation, supervision, resources, writing – review and editing.

## Funding

The authors extend their appreciation to the Researchers Supporting Project number: Health and Health Technology Project of Hubei Provincial (WJ2025Q041), Natural Science Foundation of Hubei Province (2024AFD318), General Program of China Postdoctoral Science Foundation (2024M750854), National Famous Veteran Traditional Chinese Medicine Expert Inheritance Studio Construction Project in the Field of Maternal and Child Health (National TCM Personnel & Education Letter [2026] 17).

## Consent

The authors have nothing to report.

## Conflicts of Interest

The authors declare no conflicts of interest.

## Data Availability

The data that support the findings of this study are openly available in Gene Expression Omnibus (GEO) at https://www.ncbi.nlm.nih.gov/geo/query/acc.cgi?acc=GSE328358, reference number GSE328358.
